# Insights into the evolution and fruit color change‐related genes of chromosome doubled sweet cherry from an updated complete T2T genome assembly

**DOI:** 10.1002/imo2.13

**Published:** 2024-06-30

**Authors:** Xin Zhang, Xuwei Duan, Jing Wang, Xiaoming Zhang, Guohua Yan, Chuanbao Wu, Yu Zhou, Kaichun Zhang

**Affiliations:** ^1^ Institute of Forestry and Pomology, Beijing Academy of Agriculture and Forestry Sciences Beijing People's Republic of China; ^2^ Cherry Engineering and Technical Research Center of the State Forestry and Grassland Administration Beijing People's Republic of China; ^3^ Key Laboratory of Biology and Genetic Improvement of Horticultural Crops (North China), Ministry of Agriculture and Rural Affairs Beijing People's Republic of China; ^4^ Beijing Engineering Research Center for Deciduous Fruit Trees Beijing People's Republic of China

## Abstract

Complete T2T genome assembly of sweet cherry. Chromosome doubled sweet cherry. Mutations that affecting fruit color related genes.

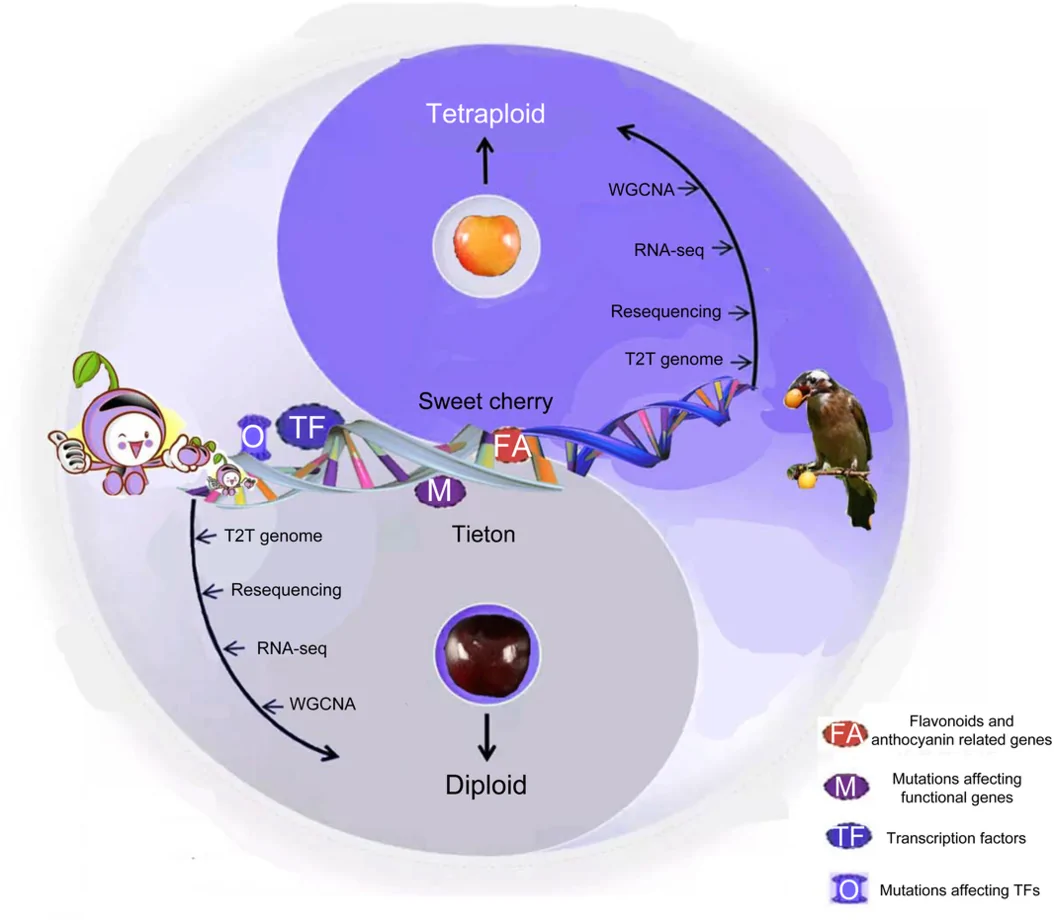

Sweet cherry (*Prunus avium* L. cv. Tieton) is a famous commercial variety for its high nutritional and healthcare value fruit, with the draft genome been assembled. At present, fundamental research on sweet cherry are mainly based on the draft genomes [1, 2, 3]. However, genome evolution based on high‐quality genome assembly that provides large collinear regions has never been conducted. Meanwhile, the research on genomic and phenotypic changes of chromosome doubled variety remain to be carried out. In view of the lack of high‐quality genomic information to promote sweet cherry genomic research, we used Tieton to assemble the complete 341.62 Mb telomere‐to‐telomere (T2T) genome after high‐throughput chromosome conformation capture (Hi‐C) correction. We used advanced sequencing technology third−generation circular consensus sequencing (CCS) and Hi‐C to assemble a high‐quality genome. Furthermore, the new characteristics of evolution and fruit color change‐related genes of chromosome‐doubled sweet cherry were investigated using this T2T genome. We analyzed repetitive sequences and coding genes, and studied the evolutionary relationship and genetic variations between sweet cherries and other *Rosaceae* plants. On the basis of RNA‐seq and resequencing analysis, we were able to identify differentially expressed genes (DEGs) in ripening fruits and mutations that occurred when the chromosome of sweet cherry were doubled, revealing the changes of potential fruit color change genes after chromosome doubling. Taken together, our study presents the latest up‐to‐date complete T2T genome of Tieton, shedding new insights into genomic evolution and alterations during chromosome doubling, and potential changes of fruit color genes.

## 1 RESULTS AND DISCUSSION

### Genome assembly and annotation

**Figure 1:**
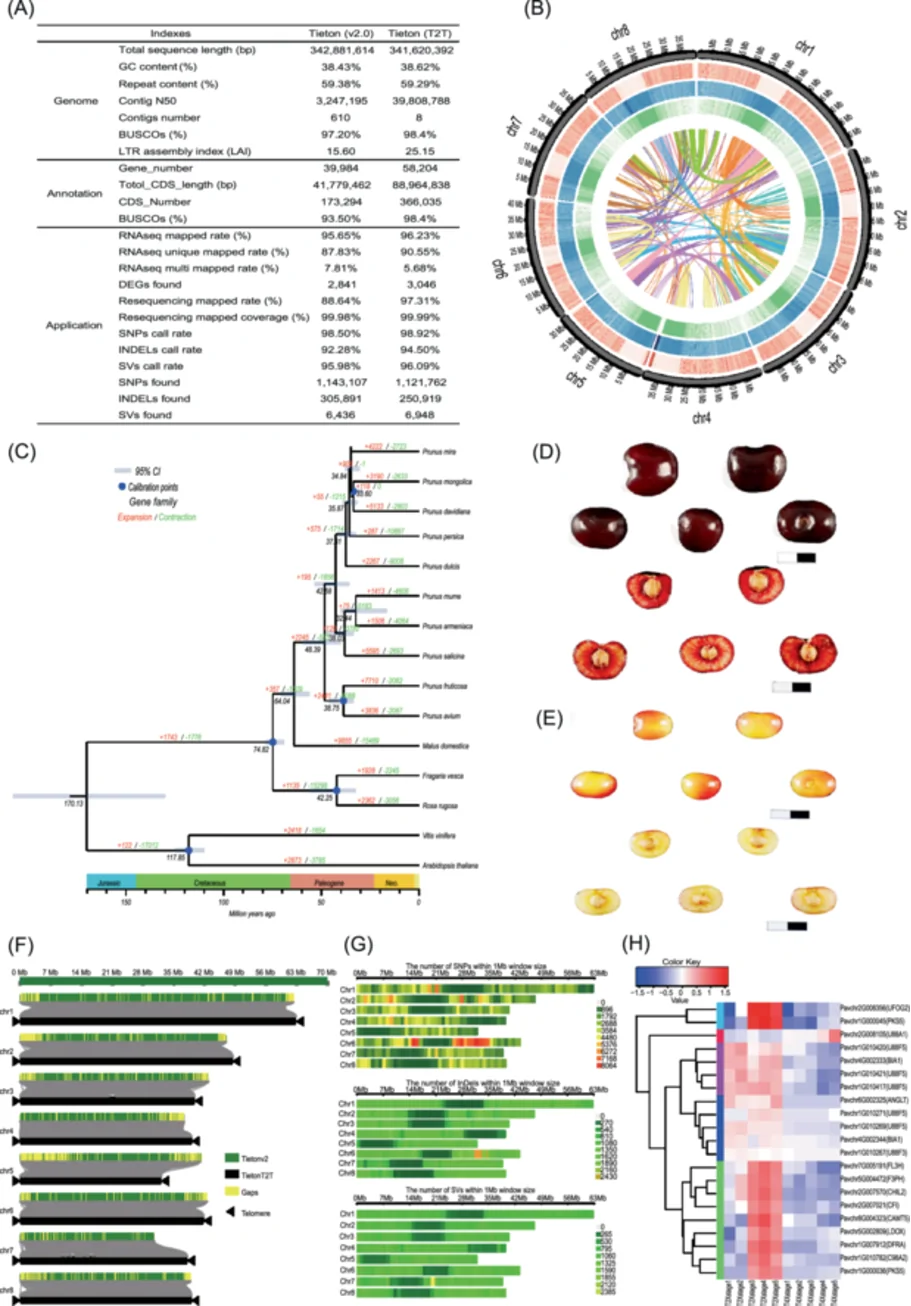
The latest complete T2T genome assembly of Tieton. (A) Tieton genomes data. The genomes of the Tieton of “v2.0” and “T2T” were compared; (B) circular plot of Tieton chromosomes. From the out to the inner: The eight chromosomes in proportion to their actual length; the genes density; genomic GC content; total repeats; genome‐wide collinear blocks; (C) evolutionary tree showing the relationships among 15 species with differentiation time; (D) cross section of ripening stage fruits of diploid (T2X) Tieton, the toolbar is 2 cm; (E) cross section of ripening stage fruits of tetraploid (T4X) Tieton, the toolbar is 2 cm; (F) collinearity between the Tieton “v2.0” and “T2T” genomes. Collinear regions are shown by gray lines. All the gap regions closed are shown as yellow blocks; (G) Mutation distribution and density of the identified mutations in T4X Tieton; (H) Heatmap of the mutation influenced DEGs (fruits color change related genes, genes names in parentheses).

To assembly the genome, 18.46 Gb (50.02×) MGISEQ reads, 20.94 Gb (61.32×) PacBioHiFi reads, and 39.73 Gb (96.79×) Hi−C data were used (Table S1). After removing the contaminants, organelle sequences, and duplicated contigs, a final assembly with a total size of 341,620,392 bp and a N50 length of 39.81 Mb was generated by integrating the published genome and Hi−C data. This assembly consists of eight contigs, named Chr1−Chr8 in descending order of reported information. The BUSCO evaluation showed that the genome integrity was 98.40% (Figure 1A,B and Table S2), with about 59.29% repetitive sequences (including TEs [transposons]) (Tables S3−S4). A total of 58,204 protein‐coding genes were predicted with different pipelines, all distributed in eight chromosomes, with 56,822 (97.63%) having functional annotations (Table S5). Telomere sequences were identified in all eight chromosomes, ranging from 1448 to 3297 bp (Figure S2 and Table S6). The centromeres were predicted based on the long tandem repeat sequence and Hi−C data (Figures S2−S3). In addition, the centromere region usually lacks genes, and the type of repeats varied in different centromeres, such as LTR (long terminal repeats)/Copia or Gypsy repeats (Figures S4−S5). All the previous reported 2152 gaps were well closed in this T2T genome (Figure 1A,F). Further, the new genome found 205 new DEGs, 512 increased structure variants (SVs) and 9.55 increased LAI (LTR assembly index value) index, comparing with the previous published genome (Figure 1A) [2], indicating the high quality of this new genome (Table S2).

### Comparative genomic analysis

With this high‐quality genome, a rooted tree was constructed (Figure 1C). Based on molecular clock analysis, *Prunus* diverged from *Malus domestica* in 64.04 million years ago (Mya) and other species evaluated gradually from *Prunus avium* approximately 48.39 Mya (Figure 1C). This large time span (95% confidence interval) was owing to the low quality of other genomes and no fossil calibration information, and we inferred a relatively recent divergence when comparing with other species [3]. We further conducted expansion and contraction analysis and discovered 3836 expanded and 2087 contracted families in *Prunus avium* (Figures S6−S7) compared with 1506 expanded and 4054 contracted families in *Prunus armeniaca* (Figure 1C). No recent whole genome duplication events were found in the genome of *Prunus avium*, which is consistent with the 4DTv curves, while the slightly upper curve may due to the gene family expansion (Figure S8−S9) [3].

The gene family distribution of the 15 analyzed species (Figure 1C) was shown in Figures S10−S11 and we found 322 unique gene families in the new genome. Collinearity analysis showed that the percentage of collinear genes was high, indicating the close relationship in *Prunus*. Several collinear genes that played roles in fruit ripening were detected in 10 *Prunus* species in Figure 1C (Figure S12), thus we further analyzed the fruit ripening‐related genes.

### Chromosome doubling, transcriptome, and mutation analysis

One of the most urgent tasks in sweet cherry cultivation is to create tetraploid sweet cherry germplasm, by which the disease resistance, flavor, and color genes can be introduced from diverse homoploid Chinese cherry resources [3]. Therefore, studying chromosome doubled genomic changes not only helps to identify trait‐related genes but also has important practical significance for creating new sweet cherry germplasm. We first obtained resequencing and transcriptomic data for Tieton diploid (T2X) and tetraploid (T4X) individuals, which were created and observed for more than 10 years (Figures 1D,E and S1; Table S1). Using this data set and our new gap‐free genome (Figure 1F), 3046 DEGs (1718 down and 1328 up) were found between fruits of the T2X and T4X (Figures 1A and S13−S14). The key pathways that these DEGs are involved were further investigated by the time series, WGCNA, and KEGG analysis (Figures S15−S21). To explore to what extent the tetraploid mutations influence the found flavonoids and anthocyanin pathways, 1,121,762 high‐quality SNPs, 250,919 INDELs and 6948 SVs were identified when comparing T4X data with the new T2X genome (Figure 1G and Table S7), with influenced (silenced) 85.71% of the flavonoids and anthocyanin DEGs observed by chromosome doubling site spectrum (Figure 1H and Table S8). These newly found mutations that influenced coexpressed transcription factors (Table S9) will provide potentially new targets for further elucidating color change mechanism of chromosome‐doubled sweet cherries and fill the blank of genomic change mechanism of chromosome‐doubled plants [4].

## AUTHOR CONTRIBUTIONS

**Xin Zhang:** Conceptualization; writing original draft, methodology, writing review and editing; visualization, investigation. **Xiaoming Zhang, Guohua Yan,** and **Yu Zhou:** Visualization; writing review and editing, project administration, investigation. **Kaichun Zhang, Xuwei Duan, Jing Wang,** and **Chuanbao Wu:** Visualization; writing review and editing, project administration, investigation.

## CONFLICT OF INTEREST STATEMENT

The authors declare no conflict of interest.

## ETHICS STATEMENT

No animals or humans were involved in this study.

## Supporting information


**Figure S1:** Flow cytometry identification of induced tetraploid sweet cherry.
**Figure S2:** Telomeres detection map.
**Figure S3:** Hi‐C heatmap of chromosome interactions.
**Figure S4:** The centromeres detection map.
**Figure S5:** Density map of LTR/Copis, LTR/Gypsy, and protein coding genes along chromosomes.
**Figure S6:** The KEGG analysis of the expanded genes families.
**Figure S7:** The KEGG analysis of the contracted genes families.
**Figure S8:** Synonymous substitutions per site (Ks).
**Figure S9:** Fourfold synonymous third‐codon transversion rate (4DTv) distribution.
**Figure S10:** Genes families distribution and unique genes families in the 15 species.
**Figure S11:** The KEGG analysis of the unique genes families.
**Figure S12:** Collinearity diagram including the 10 *Prunus* species.
**Figure S13:** Genes expression MA map.
**Figure S14:** The KEGG analysis of the differential genes.
**Figure S15:** The time series (MFUZZ) analysis of the genes in the T2X samples.
**Figure S16:** The time series (MFUZZ) analysis of the genes in the T4X samples.
**Figure S17:** The WGCNA analysis of the genes**.**
**Figure S18:** The analysis of the modules in the WGCNA analysis results.
**Figure S19:** The KEGG analysis of the biseque4 genes in the WGCNA analysis results.
**Figure S20:** The KEGG pathways analysis of the flavonoid DEGs.
**Figure S21:** The KEGG pathways analysis of the anthocyanin DEGs.

**Table S1:** Statistics of sequencing data for sweet cherry (*Prunusavium*cv. Tieton).
**Table S2:** Quality statistics of genome assembly and annotation.
**Table S3:** Statistics of the TE repetitive sequences annotated in sweet cherry (*Prunusavium* cv. Tieton) T2T genome.
**Table S4:** Statistics of the intact TE repetitive sequences and genes annotated in chromosome.
**Table S5:** Statistics of the functional annotated genes in the genome.
**Table S6:** Statistics of the telomeres and centromeres sequences in the genome.
**Table S7:** Statistics of the mutations types in the T4X genome.
**Table S8:** Statistics of the mutations that affecting the flavonoid and anthocyanidin genes in the T4X.
**Table S9:** Statistics of the mutations influenced transcription factors that co‐expressed with the flavonoid and anthocyanidin genes in the T4X.

## Data Availability

All the data for this project is in the CNGBdb under BioProject accession: https://db.cngb.org/search/project/CNP0004619/ and http://www.cherries.org.cn. Supplementary materials (methods, figures, tables, graphical abstract, slides, videos, Chinese translated version and update materials) may be found in the online DOI or iMeta Science http://www.imeta.science/imetaomics/.
