# Supplementary material for: Insights into the evolution and fruit color change‐related genes of chromosome doubled sweet cherry from an updated complete T2T genome assembly
Source: IMetaOmics. 2024 Jun 30;1(1):e13. doi: 10.1002/imo2.13 (PMC12806528; doi:10.1002/imo2.13)
Supplement: Supplementary file 1 — Figure S1: Flow cytometry identification of induced tetraploid sweet cherry. Figure S2: Telomeres detection map. Figure S3: Hi‐C heatmap of chromosome interactions. Figure S4: The centromeres detection map. Figure S5: Density map of LTR/Copis, LTR/Gypsy, and protein coding genes along chromosomes. Figure S6: The KEGG analysis of the expanded genes families. Figure S7: The KEGG analysis of the contracted genes families. Figure S8: Synonymous substitutions per site (Ks). Figure S9: Fourfold synonymous third‐codon transversion rate (4DTv) distribution. Figure S10: Genes families distribution and unique genes families in the 15 species. Figure S11: The KEGG analysis of the unique genes families. Figure S12: Collinearity diagram including the 10 Prunus species. Figure S13: Genes expression MA map. Figure S14: The KEGG analysis of the differential genes. Figure S15: The time series (MFUZZ) analysis of the genes in the T2X samples. Figure S16: The time series (MFUZZ) analysis of the genes in the T4X samples. Figure S17: The WGCNA analysis of the genes. Figure S18: The analysis of the modules in the WGCNA analysis results. Figure S19: The KEGG analysis of the biseque4 genes in the WGCNA analysis results. Figure S20: The KEGG pathways analysis of the flavonoid DEGs. Figure S21: The KEGG pathways analysis of the anthocyanin DEGs. [file IMO2-1-e13-s001.docx]

Supporting information to

**Insights into the evolution and fruit color change related genes of chromosome doubled sweet cherry from an updated complete T2T genome assembly**

Xin Zhang^1, 2, 3, 4^, Xuwei Duan^1, 2, 3, 4^, Jing Wang^1, 2, 3, 4^, Xiaoming Zhang^1, 2, 3, 4^ , Guohua Yan^1, 2, 3, 4^, Chuanbao Wu^1, 2, 3, 4^, Yu Zhou^1, 2, 3, 4^, Kaichun Zhang^1, 2, 3, 4^

**Running title：**T2T genome and fruit color related genes of chromosome doubled sweet cherry

1 Institute of Forestry and Pomology, Beijing Academy of Agriculture and Forestry Sciences, Beijing 100093, P.R.China;

2 Cherry Engineering and Technical Research Center of the State Forestry and Grassland Administration, Beijing 100093, P.R.China;

3 Key Laboratory of Biology and Genetic Improvement of Horticultural Crops (North China), Ministry of Agriculture and Rural Affairs, Beijing 100093, P.R.China;

4 Beijing Engineering Research Center for Deciduous Fruit Trees, Beijing 100093, P.R.China*;*

Correspondence: dxwlly@163.com (Xuwei Duan);kaichunzhang@126.com (Kaichun Zhang)*.*

**Materials and methods**

**Genome sequencing and assembly**

In our laboratory, fresh and healthy sweet cherry leaves (T2X) for genome sequencing were collected and generally named by their variety name. We immediately froze the samples in liquid nitrogen and stored them at −80 °C for DNA extraction. Using the improved CTAB method[1−4], we successfully extracted high−quality genomic DNA from the leaves and checked its quality. We then constructed a short read (150bp) library for MGIT7 sequencing and sequenced it on the MGIT7 platform to obtain reads. For PacBio sequencing and assembly, we followed the instructions to create a HiFi library and used CCS software (<https://github.com/pacificbiosciences/unalimity>) to generate consistent reads (HiFi). We used the default parameter of Hifiasm (0.16.1) to assemble the genome from HiFi sketch reads (_15kb, accuracy greater than 99%)[4−6]. Hi−C data was utilized to anchor and remove short overlapping groups, and HiC Pro v2.11.1 was used to classify the data as valid or invalid interaction pairs, retaining only valid pairs for further assembly. Lacesis was employed to cluster, sort, and orient overlapping groups, while HiFi data was used to fill gaps in the genome. Finally, we drew a heatmap of genomic interactions using HiCPlotter software [5−8]. Through these processes and the HiC map, we manually improved the accuracy and completeness of the assembly. Racon and Merfin were both used for polishing the assembly[9].

**Genome annotation**

Genome annotation is the process of identifying and labeling the different parts of a genome, such as genes, regulatory sequences, and other functional elements. We used several tools to analyze the genome repeat sequences and TE elements in our study. RepeatModeler, RECON(v1.08) [10], and RepeatMask were used to annotate repeat sequences, while the TE library of EDTA[4−6]was used for TE element annotation. To predict protein coding genes, we employed three methods: de novo prediction, homology prediction, and RNA−seq prediction. For de novo prediction, we used Augustus, Braker2, and GlimmerHMM. Exolate(v2.2.0) was used for homology prediction. Transcriptome prediction involved de novo assembly of transcripts using Trinity and PASA, as well as genome guided assembly using HISAT2, StringTie, and Transdecoder. EVidenceModeler and PASA were used to integrate all prediction results[7−11]. Finally, gene functional annotation was performed by searching various databases including NR, NT, SWSPROT, PFAM, PANTHER, eggnog, PlantTFdb, ATH, GO, and KEGG [12].

**Genome quality assessment**

We need to assess the quality of the genome to ensure that it is accurate and reliable. In 2021, we followed Song's method to identify telomeres on chromosome[13]. The plant telomere sequence (CCCTAAA) that we found was then used in the telomere identification process developed by VGP (https://github.com/VGP/vgp−assembly). Additionally, we used the Tandem Repeats Finder (version 4.07b) [13]to identify centromere sequences from the assembled genome [13].

We checked the genomic integrity using the BUSCO database [14]. To assess genomic continuity, we calculated the length of N50. The accuracy of the genome was determined by comparing WGS sequencing data with the genome using BWA−MEM[15] and calculating alignment and coverage with qualimap2 [16]. We also compared HiFi sequencing data with the genomes using minimap2 [17−18]. Additionally, we used the LTR assembly index (LAI) of repeated sequences to evaluate the repeat regions of the assembled genome [8, 19].

**Comparative genomic analysis methods**

We used OrthoFinder v2.4 [4−6] software to organize the protein sequences of 15 species into families, using the diamond alignment method with an e−value of 0.001. Then, we annotated the obtained gene families using the Panther v15 database. Next, we analyzed the gene copy numbers of each gene family in each species and performed GO and KEGG enrichment analyses on the unique gene families using clusterProfiler v3.14.08167 [4−6]. We created an evolutionary tree using a single copy gene sequence as input for IQ−TREE v1.6.1182. To compare the sequences of each single copy gene family, we used MAFFT v7.20583 with the parameter −localistry maximum 1000, and then filtered out regions with poor sequence alignment or significant differences using gBlocks v0.91b84 with the parameter −B5=h [4−6]. We connected all well−aligned gene family sequences of each species to obtain super genes, and then used the IQ−TREE detection tool Finder model to construct an evolutionary tree using the maximum likelihood (ML) method with a bootstrap of 1000 [4−6]. The divergence time was calculated using the PAML v4.9i software package MCMCTree 9i with the model plants *Arabidopsis thaliana* and *Vitisvinifera* as molecular markers. Finally, we used CAFÉ v4.2 software to predict the gene families of this species that contracted and expanded relative to its ancestors [4−6].

We used ParaAT v2.054 to calculate the Ks value of the gene [4−6]to estimate the whole genome duplication (WGD) event. This was done mainly through MCScanX with parameter −m5 [4−6]. Then, we obtained all genes in the collinear blocks. The Ks and 4DTV combination method is commonly used to identify WGD events. To identify WGD events within the genome, we used WGD v1.1.1 software and custom scripts available at https://github.com/JinfengChen/Scripts [4−6].

To analyze collinearity between two species, we first used diamond v0.9.29.13087 to find gene pairs with e−value less than 1E−5 and C score greater than 0.5 (C score values were determined by JCVI software). Next, we checked if these gene pairs were located next to each other on chromosomes using the gff file. Homology analysis was then conducted with JCVI v1.11954. By performing an all against−all blast on all genes and setting a distance cutoff of 20 genes, we identified the same gene blocks. Each syngeneic block contained at least 5 gene pairs[4−6].

**Transcriptome and mutations analysis**

The chromosome doubled materials were got in the lab according to the chromosome−doubling method of plants shoots. Sweet cherry fruits samples of the diploid (T2X) and tetraploid (T4X) Tieton at the five maturity periods, namely, stage1 fruit (25 days after flowering), stage2 fruit (32 days after flowering), stage3 fruit (39 days after flowering), stage4 fruit (46 days after flowering) and stage5 fruit (53 days after flowering) [2], were collected and analyzed their RNA using RNA−seq. The data was processed on the MGIT7 platform and removed any errors and ensured accuracy. We then compared the sequences to a reference genome to evaluate the quality of the data. By looking at the gene expression differences between the two groups, we were able to identify differentially expressed genes (DEGs) using DESeq2 (v1.6.3) with specific criteria (screening criteria with FC≥2 and Error Detection Rate (FDR) <0.05). We further analyzed the DEGs using GO and KEGG databases to understand their functions and pathways[4−6].

For resequencing analysis, first, we used FASTP [20−21] software to clean the raw resequencing data from the tetraploid (T4X). Then, we compared the clean data with default parameters in BWA software to the the new diploid (T2X) Tietonreference genome. We sorted the paired data using samtools software and removed PCR duplicates from the bam file using the MarkDuplicates parameter in version 4.1.9 of GATK [20−21] to obtain the bam file. Next, we compared the SNPs, Indels, and SVs related indexes found by the two genomes respectively. We used the samtools merge parameter to merge the data from multiple sequencing of a single sample.

After that, we used the Haplotyper function of the driver module of Sentioneon software [20−22] to detect mutations. We applied the recommended standard parameters on the official website for SNPs and INDELs hard filtering, ultimately obtaining high−quality snp and indel files. Annovar was used to annotate Indel and SNP variation sites. Based on the annotation information, we defined that if the SNP is a non−synonymous mutation located on the exon, it is considered to affect the expression of the gene. If an indel is a frameshift mutation on the gene exon, it is considered to affect the expression of the gene.

We then used the default parameters of Delly software [7] to perform SV detection on the merged Bam file and extracted the "PASS" site as the final SVs mutation site. According to their overlapping regions in the genome, SVs were divided into four categories: coding regions (overlapping with the exon regions of genes), regulatory regions (within the 2kb upstream and downstream regions of genes), introns (within the intron regions of genes), and intergenic regions (between two genes and not within the 2kb upstream and downstream regions of two genes). If SV overlaps with an exon region or an upstream or downstream 2kb region of a gene, it was considered to affect that gene [20−22].

**References**

1. Shirasawa Kenta, Isuzugawa Kanji, Ikenaga Mitsunobu, Saito Yutaro, Yamamoto Toshiya, Hirakawa Hideki, Isobe Sachiko. 2017. “The genome sequence of sweet cherry (*Prunusavium*) for use in genomics−assisted breeding.” *DNA Research* 5: 499−508.https://doi.org/10.1093/dnares/dsx020.
2. Wang Jiawei, Weizhen Liu, Dongzi Zhu, PoHong, Qingzhong Liu. 2020. “Chromosome−scale genome assembly of sweet cherry (*Prunusavium* L.) cv. tieton obtained using long−read and hi−c sequencing.” *Horticulture Research* 7(1):11.https://doi.org/10.1038/s41438−020−00343−8.
3. Sara Pinosio, Fabio Marroni, Andrea Zuccolo, Nicola Vitulo, Michele Morgante. 2020. “A draft genome of sweet cherry (*Prunusavium* L.) reveals genome−wide and local effects of domestication.” *The Plant Journal* 4: 103. https://doi.org/10.1111/tpj.14809.
4. Cheng Haoyu, Gregory T. Concepcion, Xiaowen Feng, Haowen Zhang and Heng Li. 2021.“Haplotype−resolved de novo assembly using phased assembly graphs with hifiasm.” *Nat. Methods* 18:170−175. https://doi.org/10.1038/s41592−020−01056−5.
5. Servant Nicolas, Varoquaux Nelle, Lajoie R Bryan, Viara Eric and Chong-Jian Chen. 2015. “HiC−Pro: an optimized and flexible pipeline for Hi−C data processing.” *Genome Biol* 16:259. https://doi.org/10.1186/s13059−015−0831−x.
6. Burton J.N., Adey A., Patwardhan R.P., Qiu R.L., Kitzman J.O. and Shendure J. 2013. “Chromosome−scale scaffolding of de novo genome assemblies based on chromatin interactions.” *Nat. Biotechnol* 31:1119−1125. https://doi.org/10.1038/nbt.2727.
7. Song J.M., W.Z. Xie, S. Wang, Y.X. Guo, D.H. Koo, D. Kudrna, C. Gong, Y. Huang, J.W. Feng, W. Zhang, et al. 2021. “Two gap−free reference genomes and a global view of the centromere architecture in rice.” *Mol. Plant* 14:1757−1767. https://doi.org/10.1016/j.molp.2021.06.018.
8. Akdemir K.C. and Chin L. 2015. “HiCPlotter integrates genomic data with interaction matrices.” *Genome Biol* 16:198. https://doi.org/10.1186/s13059−015−0767−1.
9. McCartney A.M., Shafin K., Alonge M., Bzikadze A.V., Formenti G., Fungtammasan A., Howe K., Jain C., Koren S., Logsdon G.A., et al. 2022. “Chasing perfection: validation and polishing strategies for telomere−to−telomere genome assemblies.” *Nat. Methods* 19:687−695. https://doi.org/10.1101/2021.07.02.450803.
10. Flynn J.M., Hubley R., Goubert C., Rosen J., Clark A.G., Feschotte C., and Smit A.F. 2020. “RepeatModeler2 for automated genomic discovery of transposable element families.” *Proc. Natl. Acad. Sci* 117:9451−9457. https://doi.org/10.1073/PNAS.1921046117.
11. Tarailo−Graovac Maja and Chen N. 2009. “Using RepeatMasker to identify repetitive elements in genomic sequences.” *CurrProtoc Bioinformatics* 4:4−10. https://doi.org/10.1002/0471250953.bi0410s05.
12. Moriya Yuki, Itoh M., Okuda S., Yoshizawa A.C. and Kanehisa M. 2007. “KAAS: an automatic genome annotation and pathway reconstruction server.” *Nucleic Acids Res* 35:182−185. https://doi.org/10.1093/nar/gkm321.
13. Huang Yongji, Ding W., Zhang M., Han J., Jing Y., Yao W., Hasterok R., Wang Z., Wang K. 2021. “The formation and evolution of centromeric satellite repeats in Saccharum species.” *Plant J* 106: 616−629. https://doi.org/10.1111/tpj.15186.
14. Manni Mosè, Berkeley Matthew R, Seppey Mathieu, Simo Felipe A., ZdobnovEvgeny M. 2021. “BUSCO update: novel and streamlined workflows along with broader and deeper phylogenetic coverage for Scoring of eukaryotic, prokaryotic, and viral Genomes.” *Mol. Biol. Evol* 38:4647−4654. https://doi.org/10.1093/molbev/msab199.
15. Li Heng and DurbinRichard. 2010. “Fast and accurate long−read alignment with Burrows−Wheeler transform.” *Bioinformatics* 26:589−595. https://doi.org/10.1093/bioinformatics/btp698.
16. Okonechnikov Konstantin, Conesa Ana and Garcı´a−Alcalde Fernando. 2016. “Qualimap 2: advanced multi−sample quality control for highthroughputsequencing data.” *Bioinformatics* 32:292−294. https://doi.org/10.1093/bioinformatics/btv566.
17. Ou Shujun, Jinfeng Chen, and Ning Jiang. 2018. “Assessing genome assembly quality using the LTR Assembly Index (LAI).” *Nucleic Acids Res* 46:e126. https://doi.org/10.1093/nar/gky730.
18. Ou S., Su W., Liao Y., Chougule K., Agda J.R., Hellinga A.J., Lugo C.S.B., Elliott T.A., Ware D. and Peterson T. 2019. “Benchmarking transposable element annotation methods for creation of a streamlined, comprehensive pipeline.” *Genome Biology* 20:1−18. https://doi.org/10.1101/657890.
19. Arang Rhie, Brian P. Walenz, Sergey Koren, Adam M. Phillippy. 2020. “Merqury: reference−free quality, completeness, and phasing assessment for genome assemblies.” *Genome Biol* 21:245. https://doi.org/10.1186/s13059−020−02134−9.
20. Li H, Handsaker B., Wysoker A., Fennell T., Ruan J., Homer N., Marth G., Abecasis G., Durbin R.,*et al.* 2009. “The sequence alignment/map format and SAMtools.”*Bioinformatics* 25: 2078−2079. https://doi.org/10.1093/bioinformatics/btp352.
21. Mckenna A., Hanna M., Banks E., Sivachenko A., Cibulskis K., Kernytsky A., Garimella K., *et al.* 2010.“The genome analysis toolkit: a MapReduce framework 288 for analyzing nextgeneration DNA sequencing data.”*Genome Res.* 20: 1297−1303. https://doi.org/10.1007/978−3−662−46703−9_5.
22. Tuskan G.A., Difazio S., Jansson S., Bohlmann J., Grigoriev I., Hellsten U. & Putnam N., et al. 2006. “The genome of black cottonwood, *Populustrichocarpa* (Torr. & Gray).” *Science* 313:1596−1604. https://doi.org/10.1126/science.1128691.


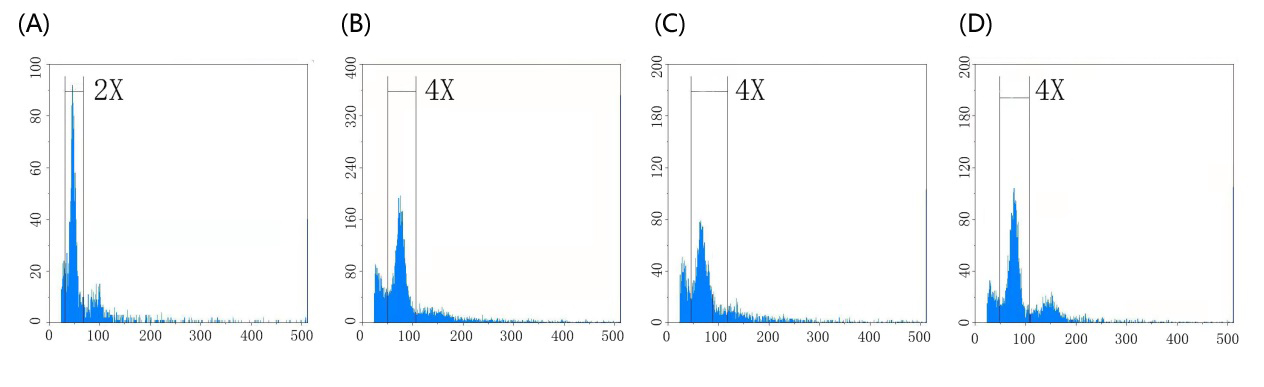


**Figure S1 Flow cytometry identification of induced tetraploid sweet cherry.**

(A) Diploid control; (B) − (D): induced tetraploid; the abscissa indicates the DNA content, and the ordinate indicates the effective cell number. 2X and 4X indicate the location of diploid and tetraploid DNA content in the abscissa


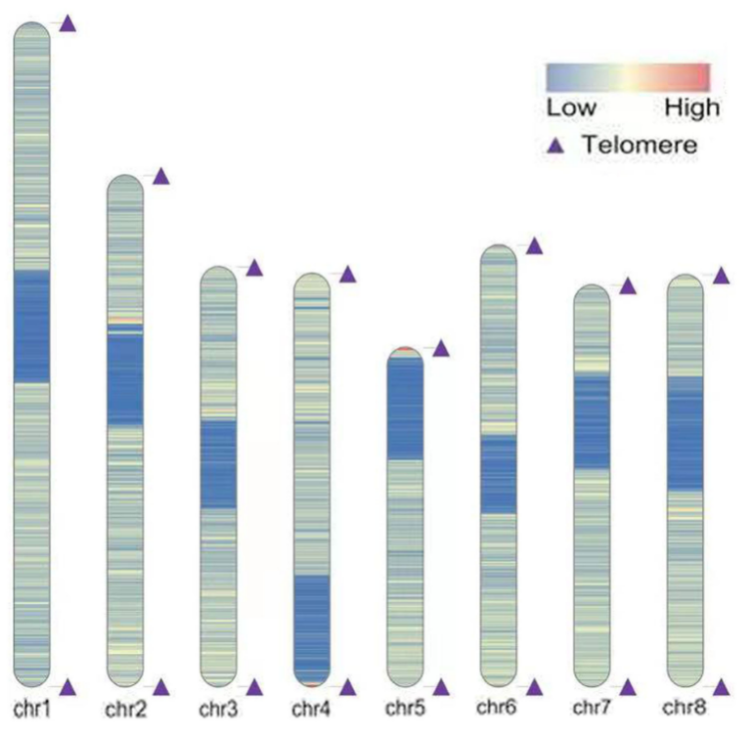


**Figure S2Telomeres detection map.**

**
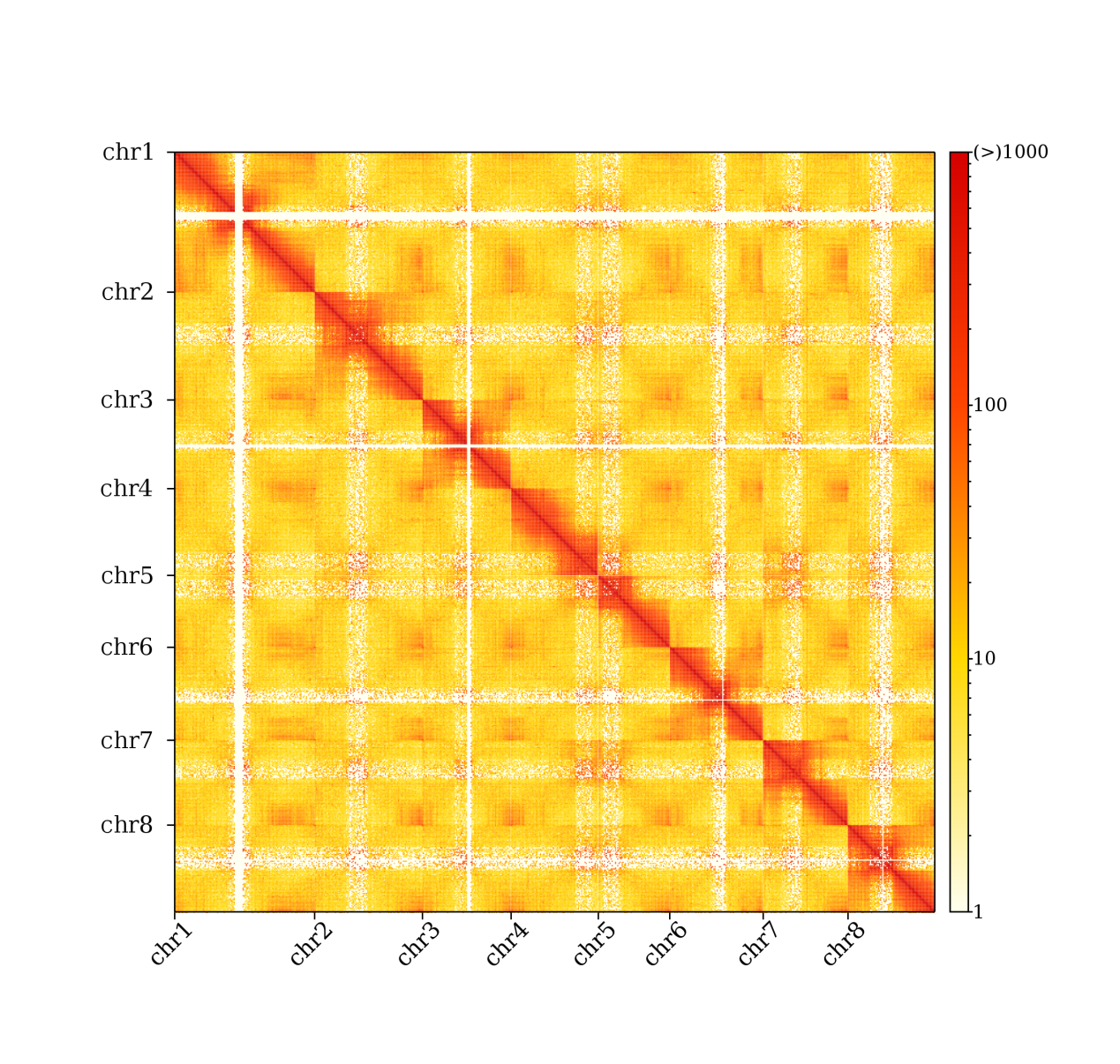
**

**Figure S3 Hi-C heatmap of chromosome interactions.**


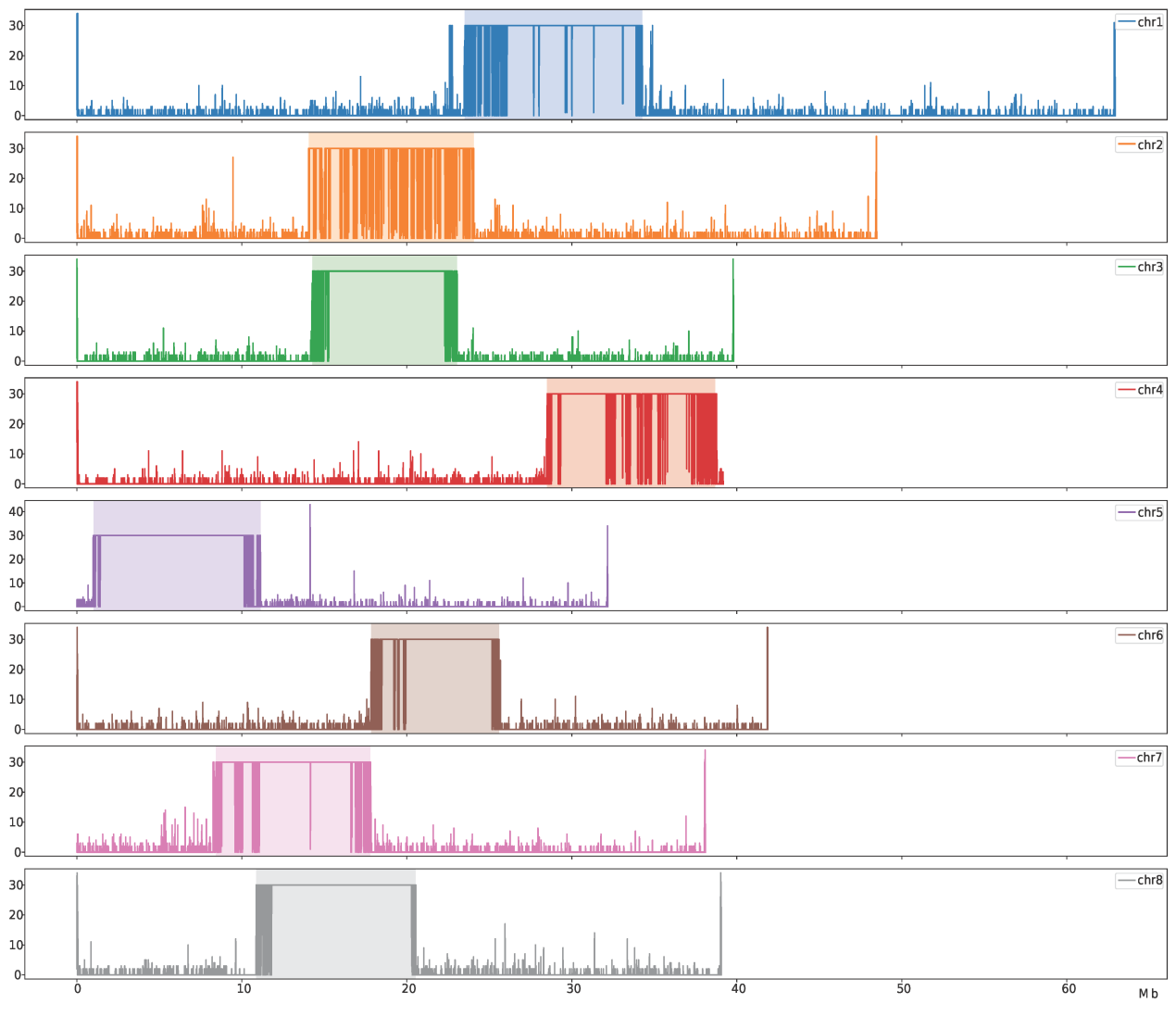


**Figure S4 The centromeres detection map.**

(The abscissa indicates the chromosomes position, and the ordinate indicates statistical value of the repetitive centromeres sequences)


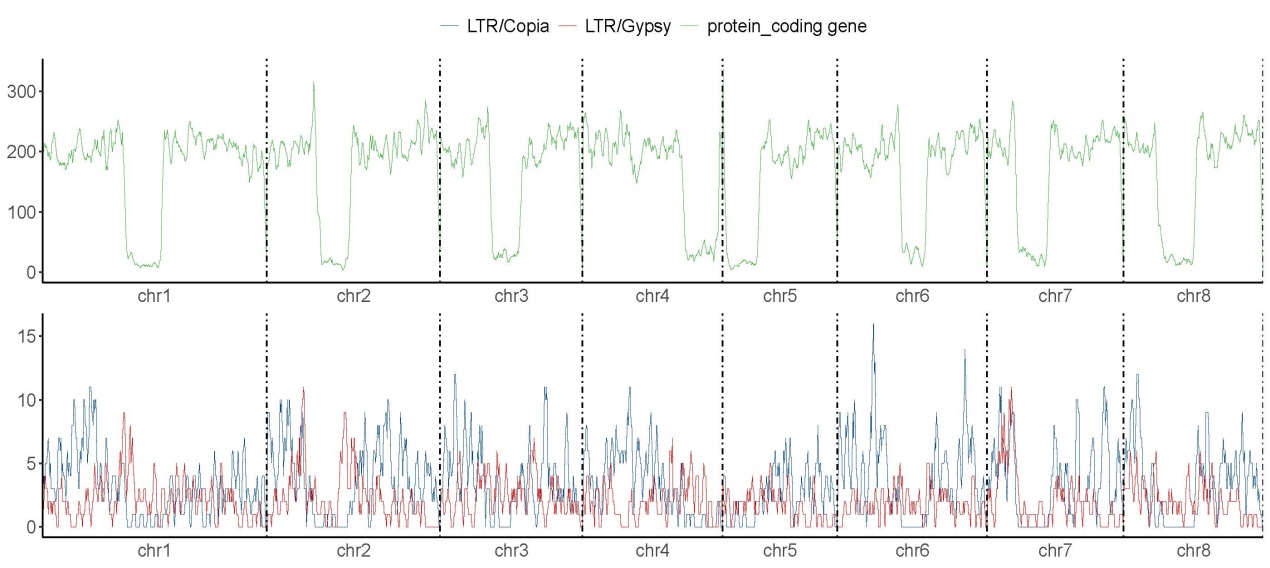


**Figure S5 Density map of LTR/Copis, LTR/Gypsy, and protein coding genes along chromosomes.**

(The abscissa indicates the chromosomes position, and the ordinate indicates statistical value of the repetitive sequences.)


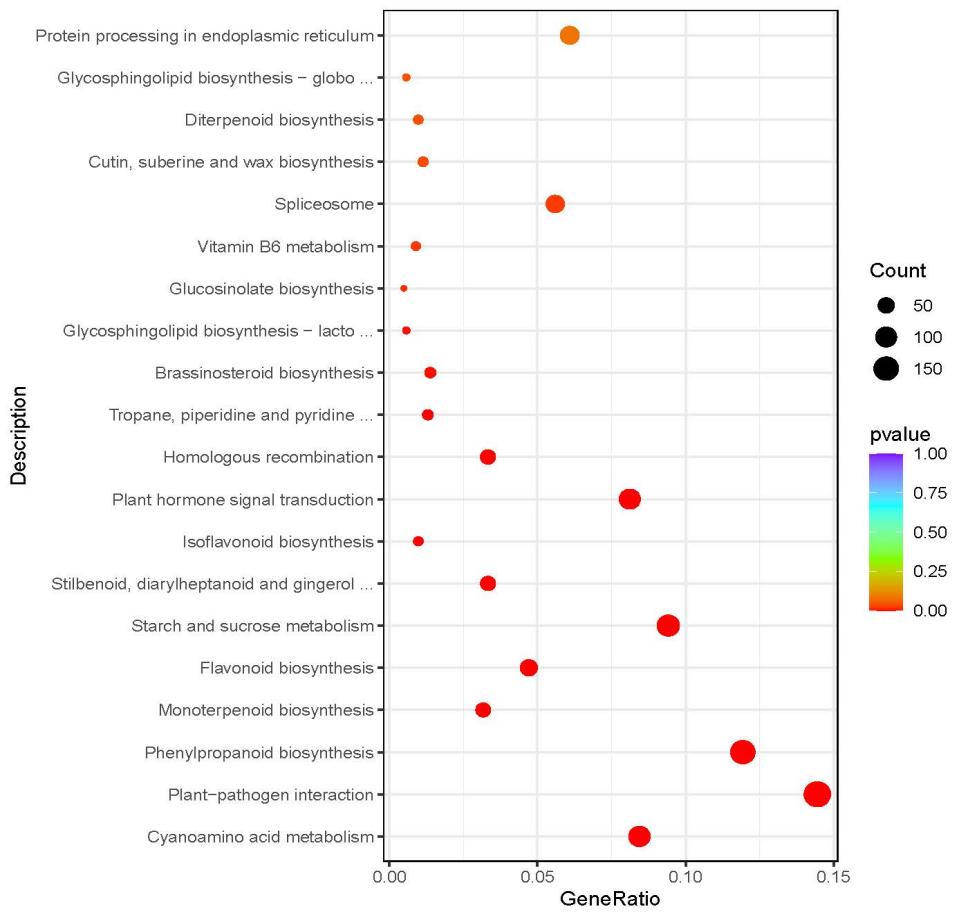


**Figure S6 The KEGG analysis of the expanded genes families.**


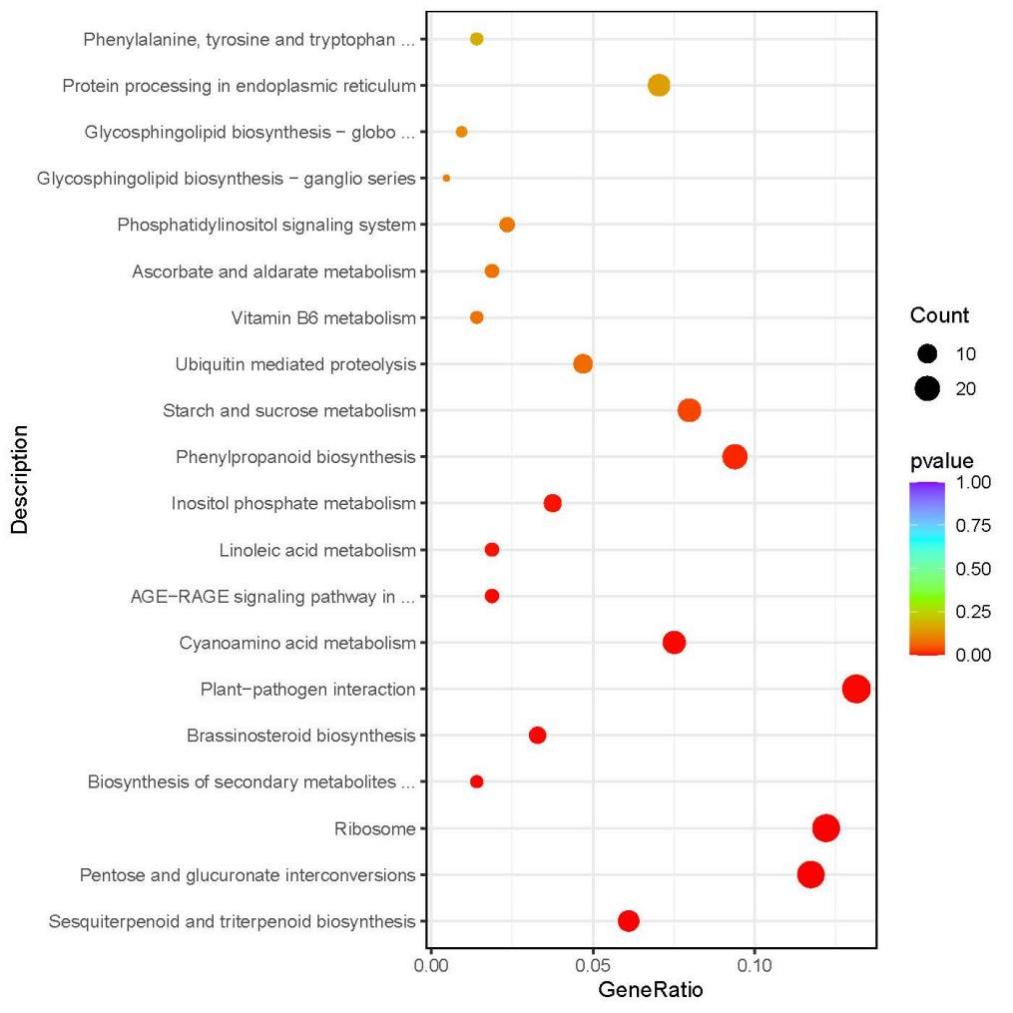


**Figure S7 The KEGG analysis of the contracted genes families.**

**
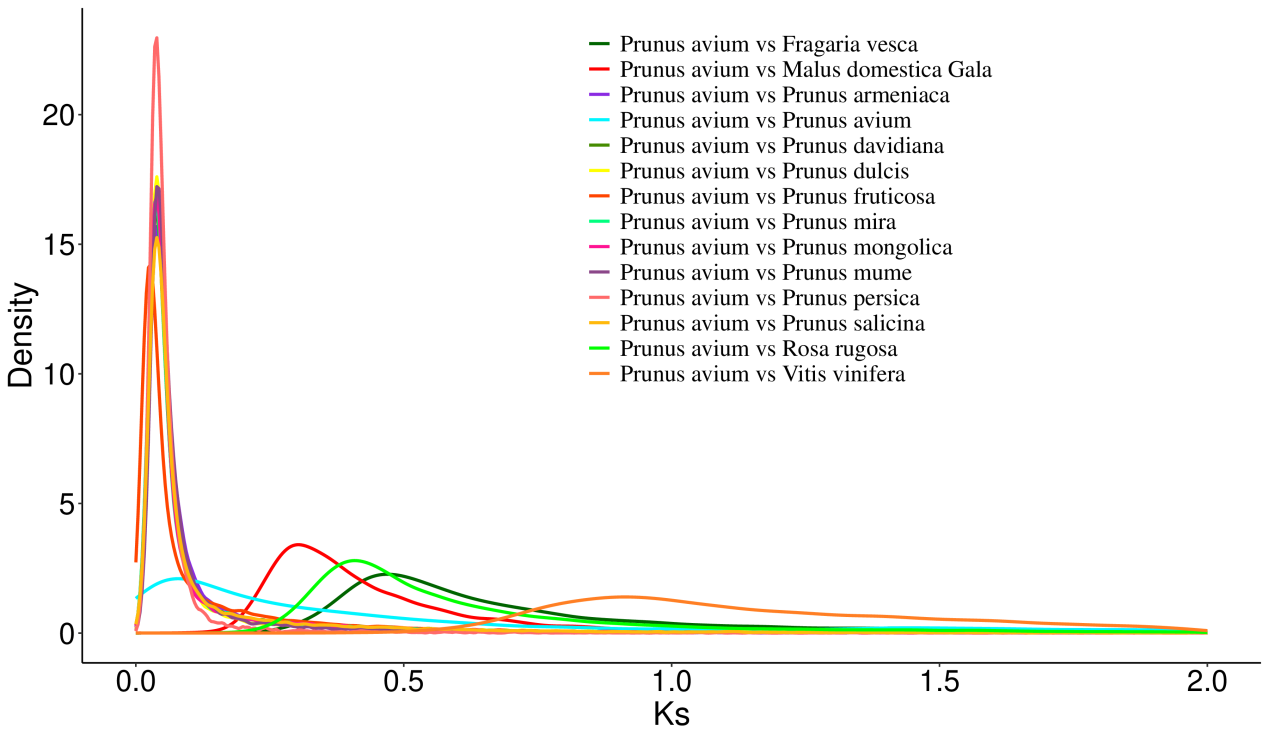
**

**Figure S8 Synonymous substitutions per site (Ks).**


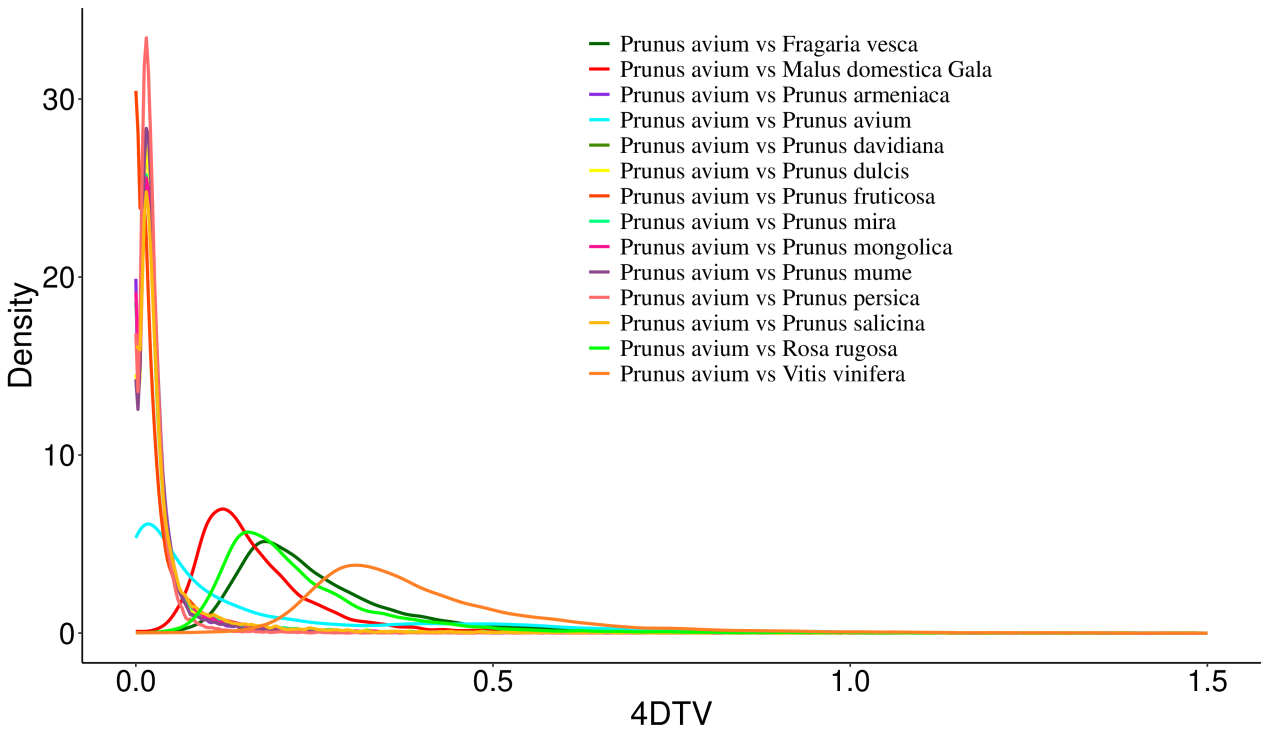


**Figure S9 Fourfold synonymous third-codon transversion rate (4DTv) distribution.**


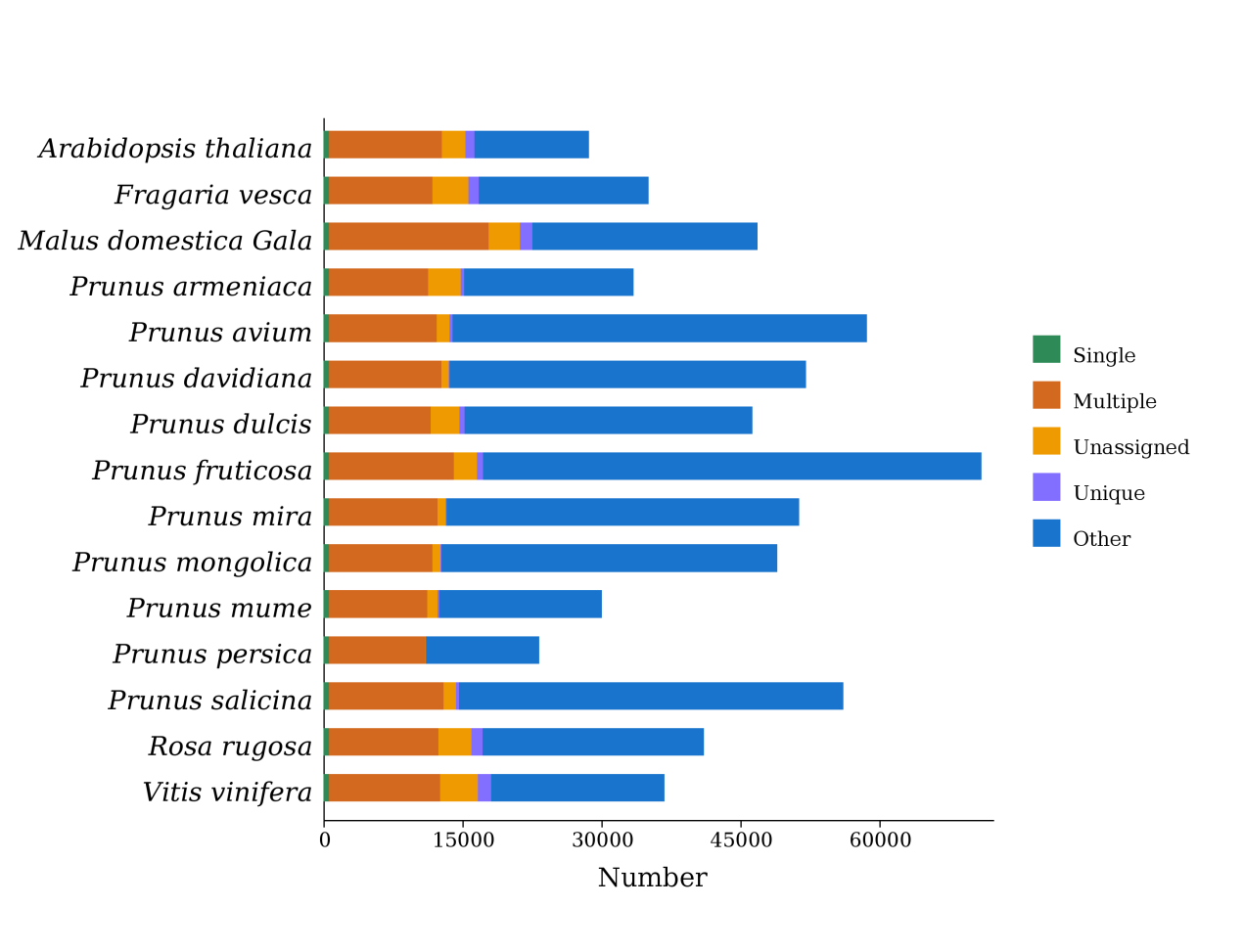


**Figure S10Genes families distribution and unique genes families in the 15 species.**


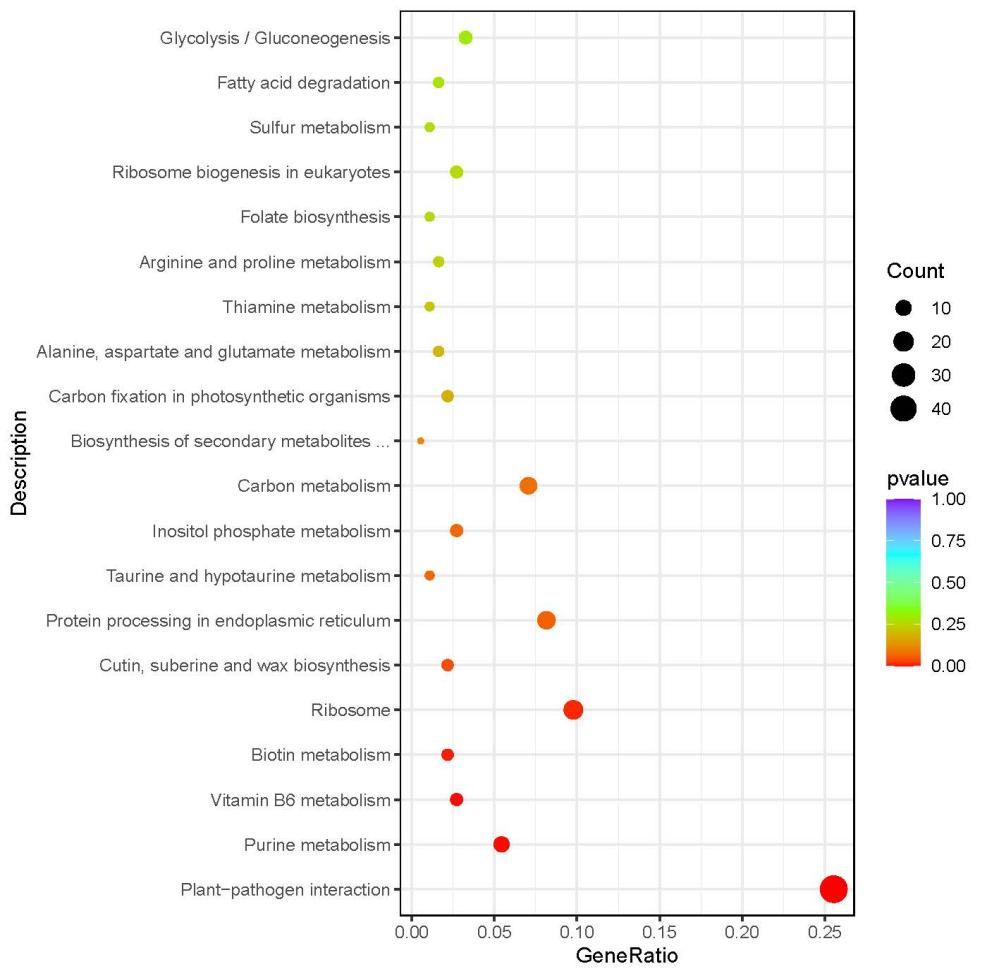


**Figure S11 The KEGG analysis of the unique genes families.**


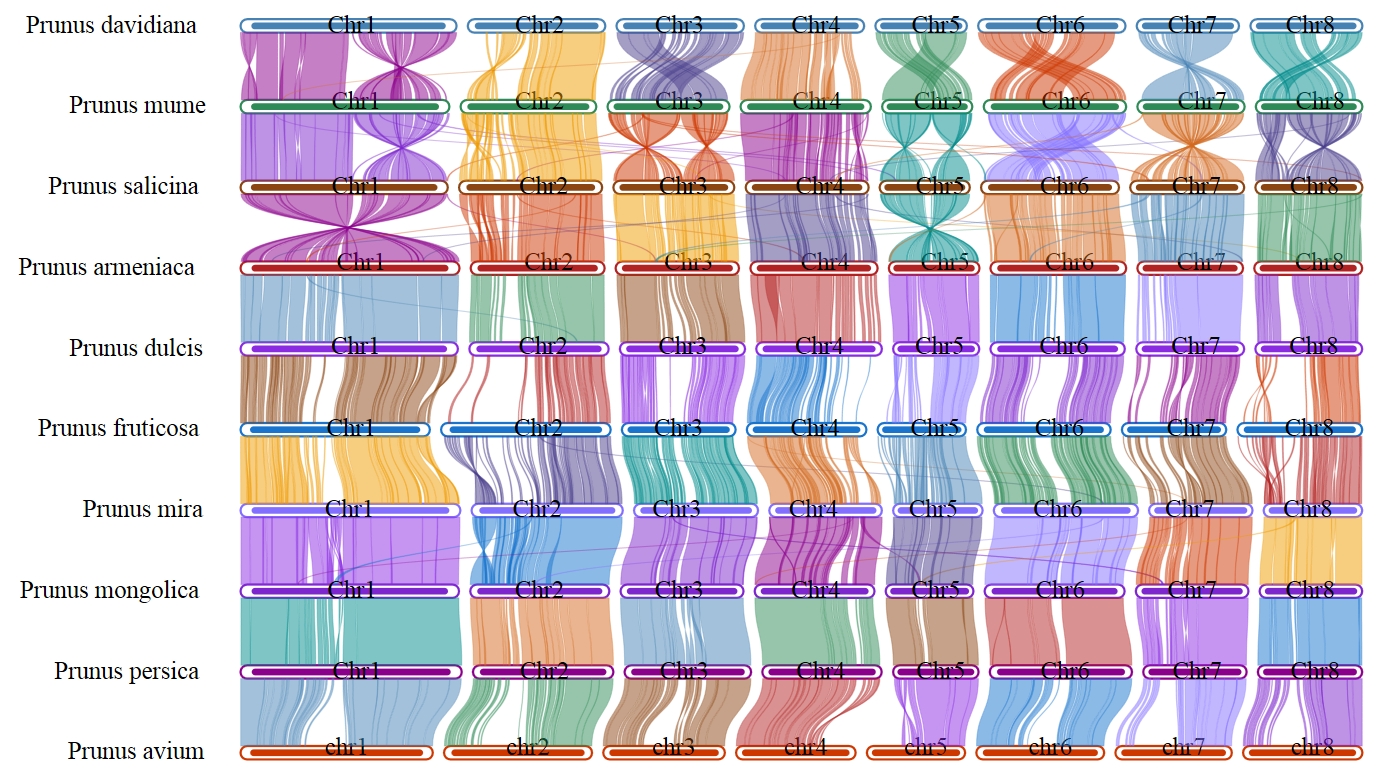


**Figure S12Collinearity diagram including the 10 *Prunus* species.**


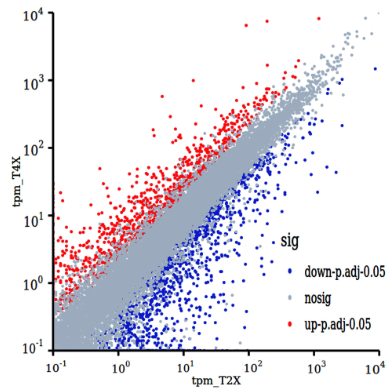


**Figure S13Genes expression MA map.**

(The same time ripenning fruits were compared between diploid (T2X) and tetraploid (T4X) Tieton.)


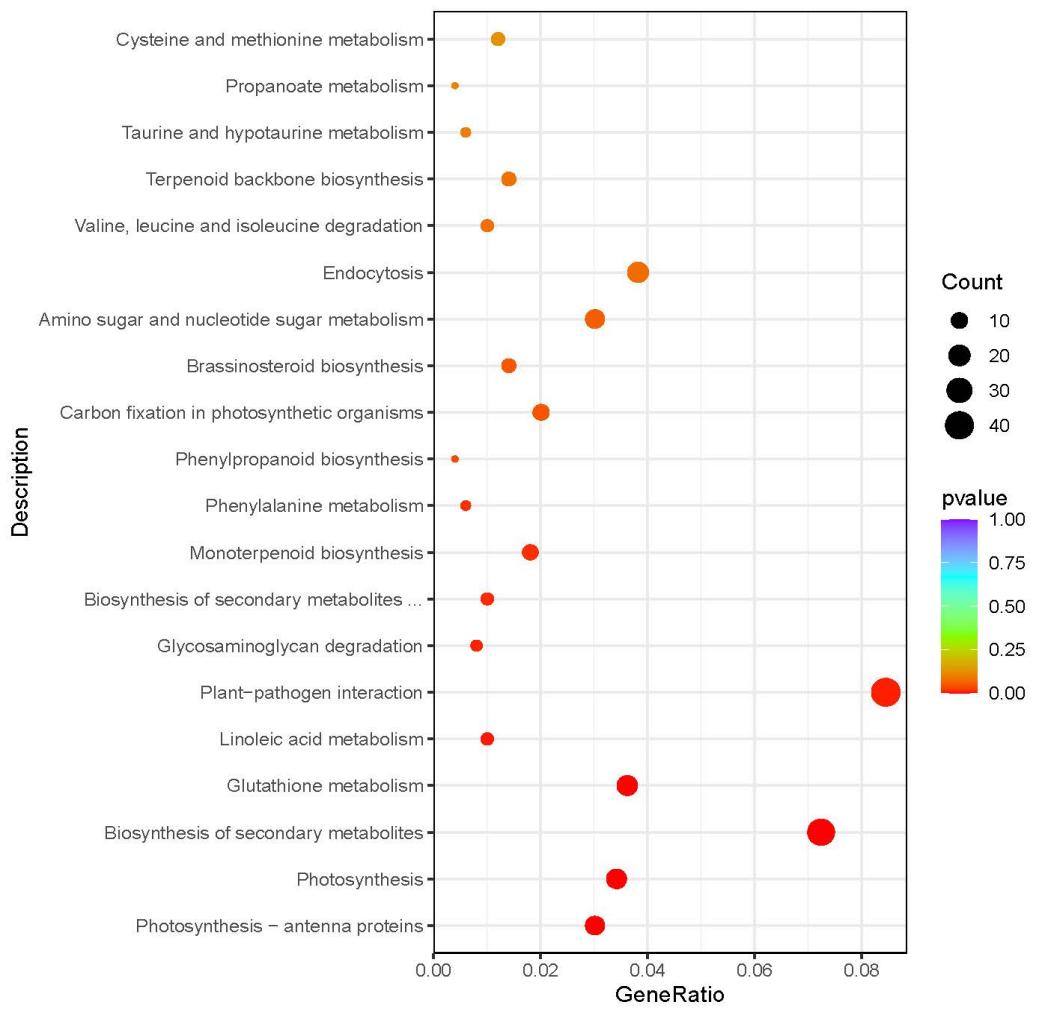


**Figure S14 The KEGG analysis of the differential genes.**


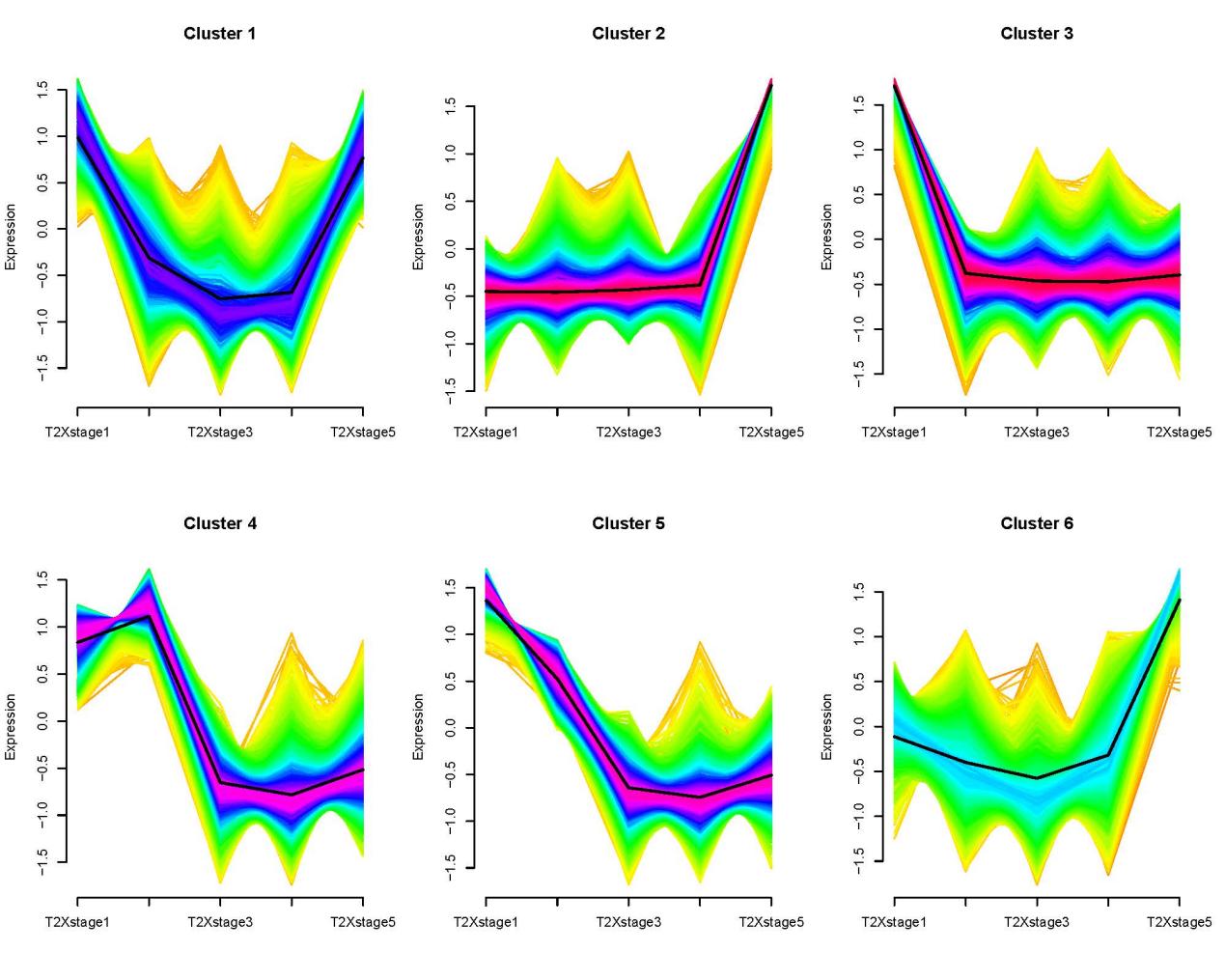


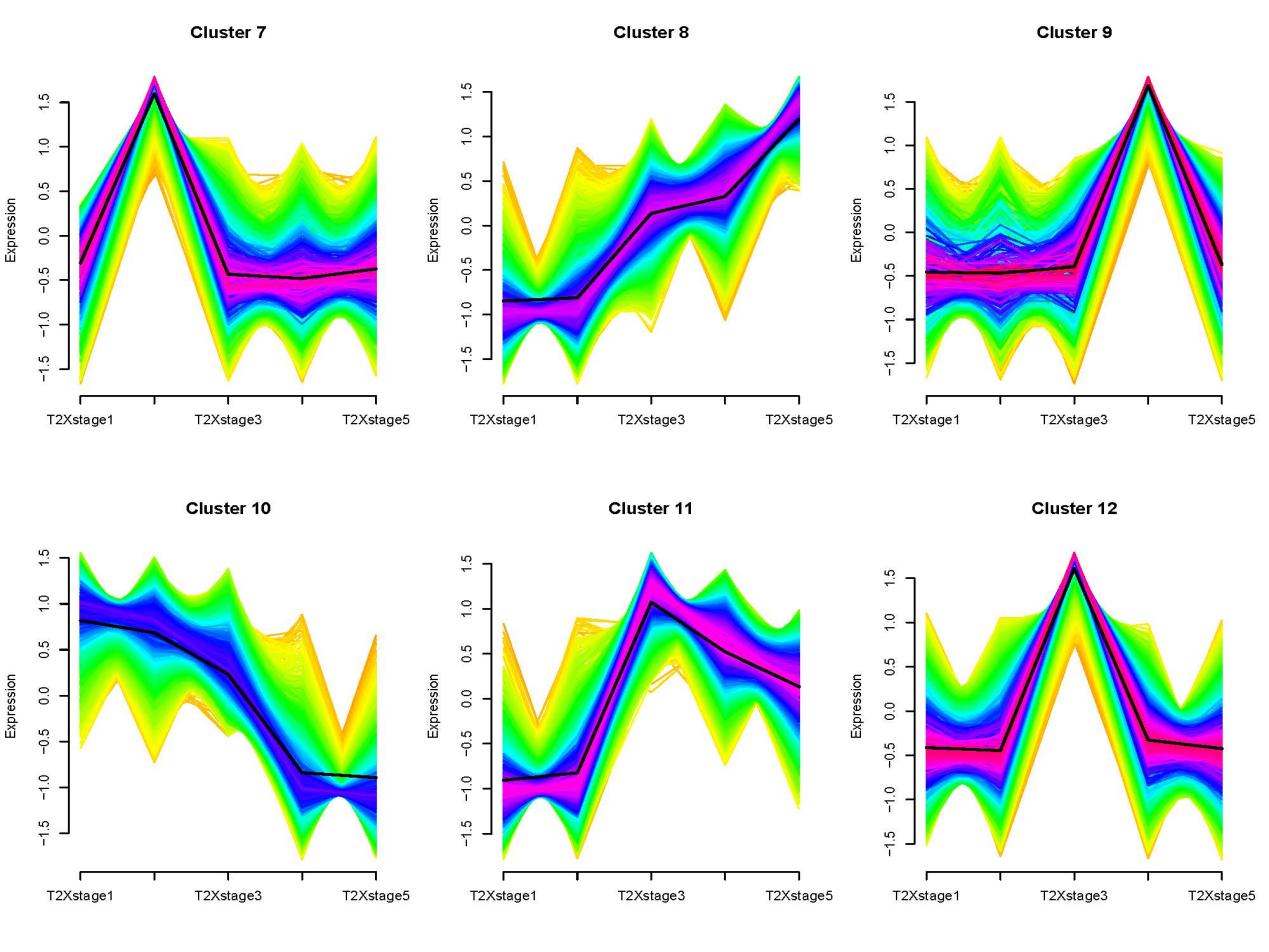


**Figure S15 The time series (MFUZZ) analysis of the genes in the T2X samples.**


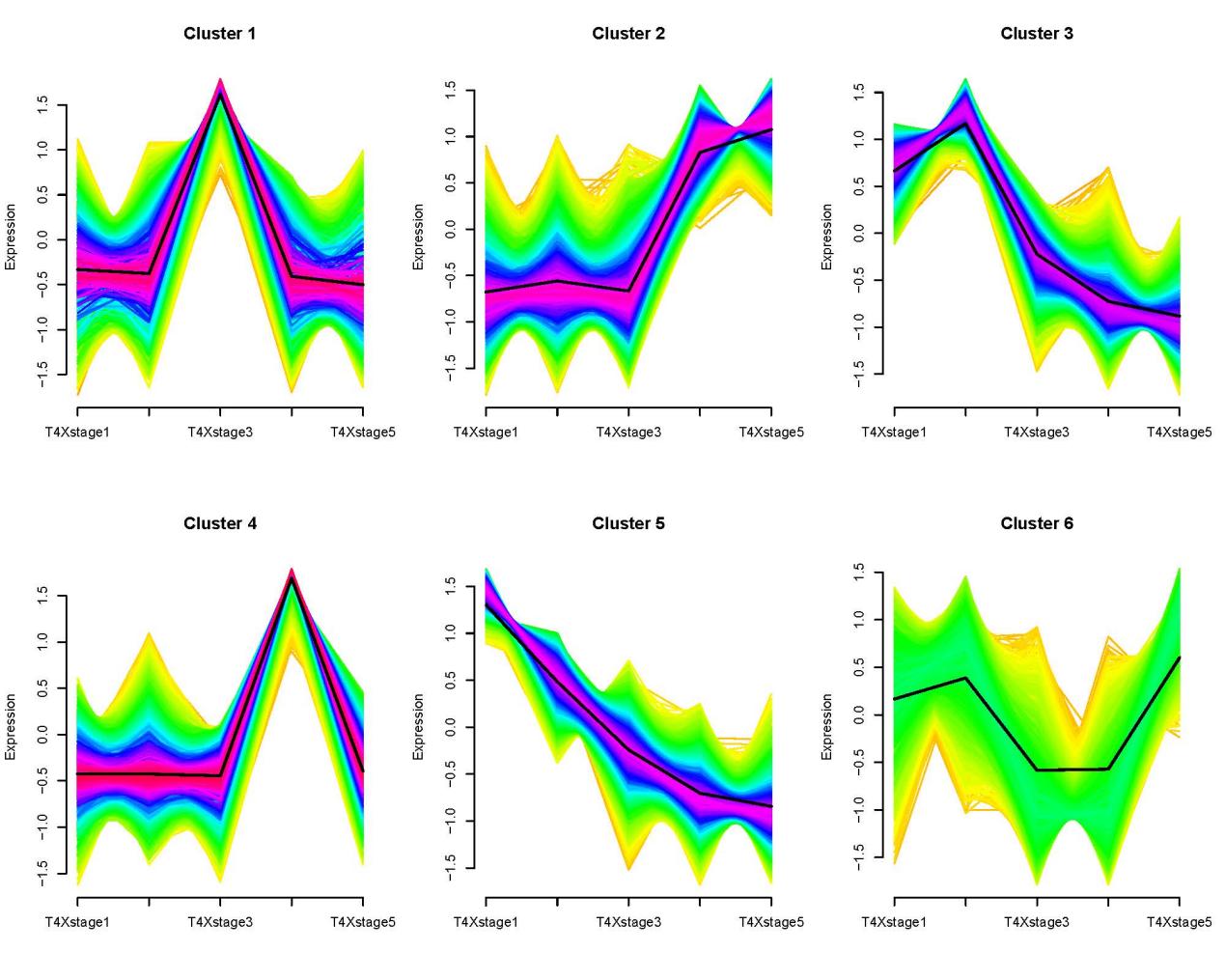


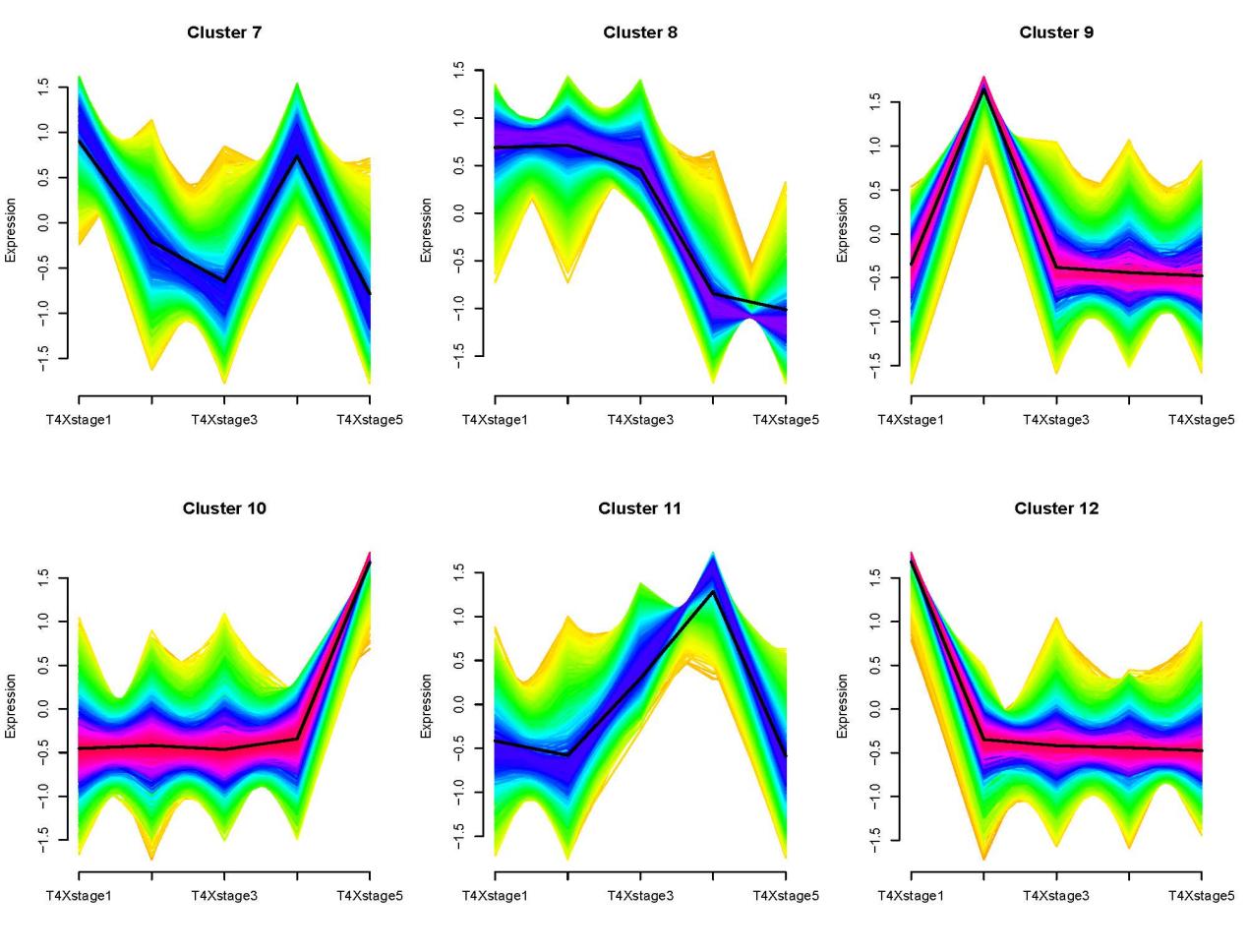


**Figure S16 The time series (MFUZZ) analysis of the genes in the T4X samples.**

**
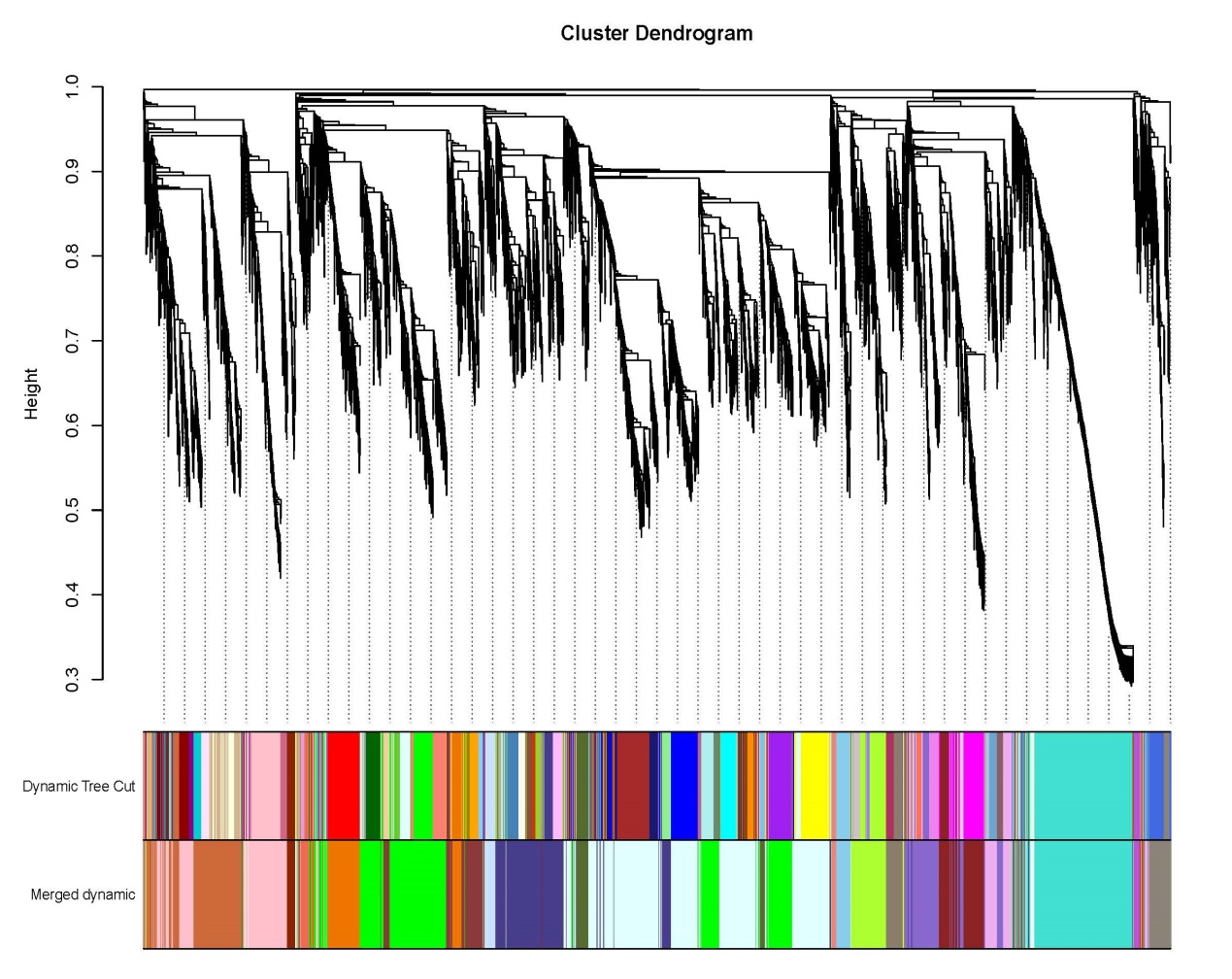
**

**Figure S17 The WGCNA analysis of the genes.**

**
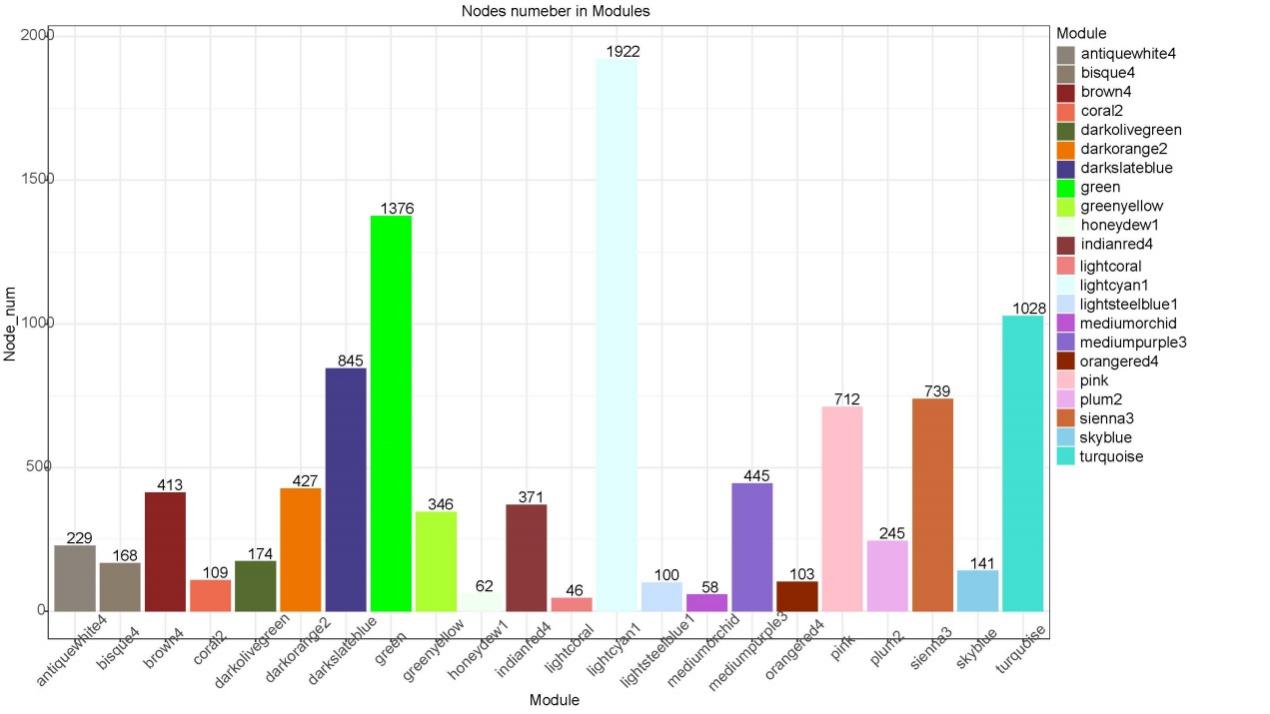
**

**Figure S18 The analysis of the modules in the WGCNA analysis results.**


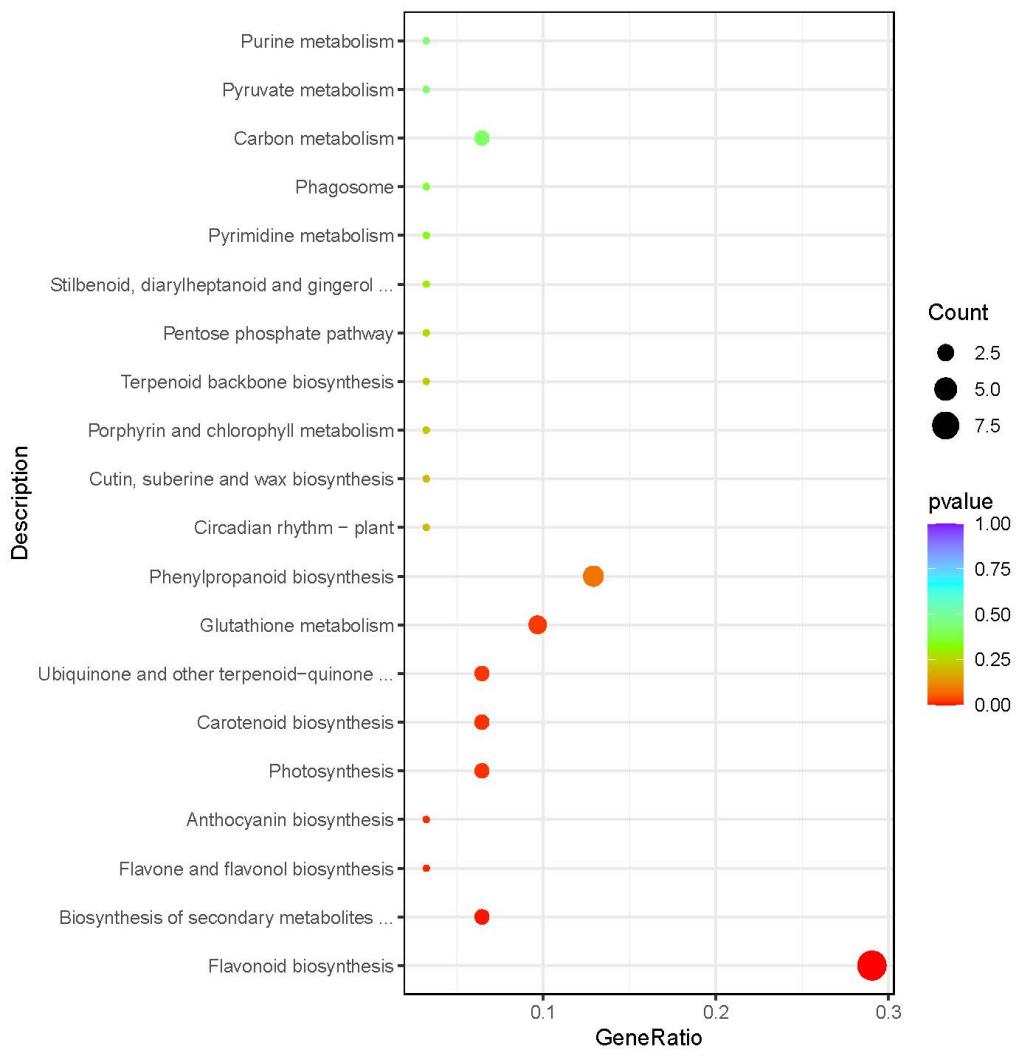


**Figure S19 The KEGG analysis of the biseque4 genes in the WGCNA analysis results.**


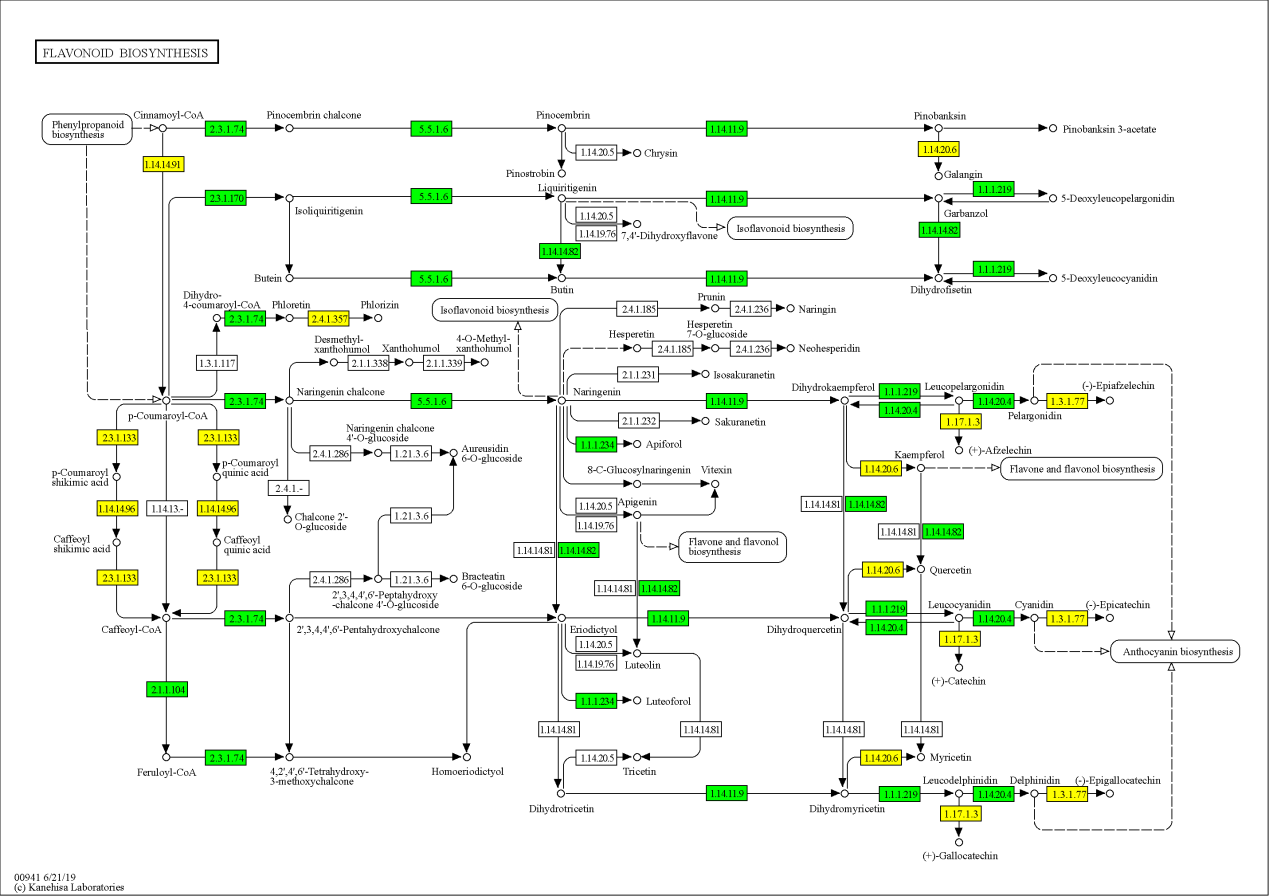


**Figure S20 The KEGG pathways analysis of the flavonoid DEGs.**

**
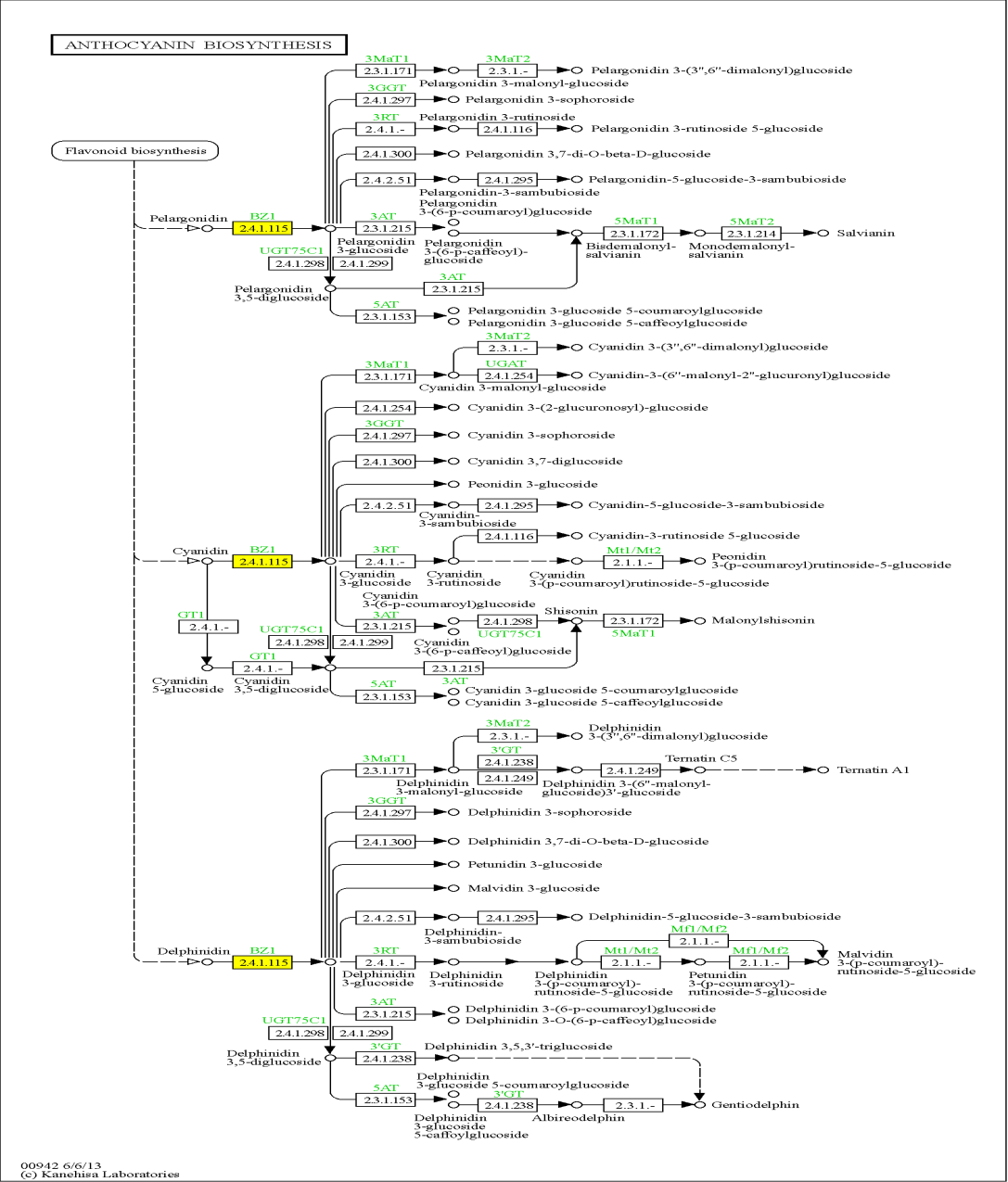
**

**Figure S21 The KEGG pathways analysis of the anthocyanin DEGs.**
